# Supplementary material for: “I’m suffering for food”: Food insecurity and access to social protection for TB patients and their households in Cape Town, South Africa
Source: PLoS One. 2022 Apr 26;17(4):e0266356. doi: 10.1371/journal.pone.0266356 (PMC9041827; doi:10.1371/journal.pone.0266356)
Supplement: S1 File — (PDF) [file pone.0266356.s001.pdf]

HEALTH SYSTEMS
RESEARCH UNIT

SUPPORTING INFORMATION FILE 1
TOPIC GUIDE FOR TB PATIENTS XHOSA
ISIKHOKELO SESIHLOKO – AMAVA EZIGULI NGE-TB

Ngaphambi kodliwano-ndlebe:

Ndazise kudliwano-ndlebe, ingcaciso emfutshane yesihloko sophononongo, injongo yophononongo "olu phononongo lujonge ukuqonda umthwalo we-TB kwezentlalo, kwezoqoqosho nokwasekhaya ngodliwano-ndlebe nezigulane malunga namava azo e-TB kunye nokubonelela ngenkxaso ye-TB. Uphononongo lukwafuna ukuqonda amava ezigulana zokhathalelo lwempilo olukhoyo kunye neenkonzo zentlalo yoluntu ukuxhasa izigulane ezine-TB."

Funda iPhepha leNgcaciso, qinisekisa imfihlo, ukusayinwa kwephepha lemvume

Chaza ubude obulindelekileyo bodliwano-ndlebe: 1 ukuya kwi-2 yeyure ubuninzi

Cacisa inkqubo yodliwano-ndlebe "Olu dliwano-ndlebe ngokwenene lumalunga nokufumana ingqiqo yokuba uziva njani xa ugula ngenxa ye-TB, impembelelo enayo ebomini bakho, kunye nendlela ofumana ngayo ukufikelela kwiinkonzo zempilo nezentlalo ukuze kuxhaswe wena."

IMBALI YE-TB

Ndicela undixelele kancinci ngawe. (Phanda ngobomi basekhaya, imisebenzi, imvelaphi, njl.njl)

Ngaba wawukhe wane-TB ngaphambili?

Yayinini lento kwaye wawuluthathile unyango ngeloxesha? (Dwelisa zonke iziqendu zangaphambili + ubude bonyango)

UMTHWALO WE-TB

Ngaba ukhe wagula kakhulu ngenxa ye-TB? Nceda uchaze, umz. Ngaba ubulele ebhedini ixesha elingakanani, uziva udiniwe, ubhityile, njl. I-TB ibuchaphazele njani ubomi bakho? (Ukuba akukho mpendulo, phononongа oku kulandelayo:

- ngaba uphose ixesha emsebenzini? Zingaphi iintsuku?
- ingaba i-TB ibe nefuthe kubudlelwane nekhaya/usapho/abahlobo bakho?
- ingaba i-TB ibe nefuthe kubomi bakho ekuhlaleni?

USULELO OLUNINZI KWI-HH

Ngubani omnye ekhayeni lakho okhe wane-TB? (Uluhlu lwawo onke amalungu osapho ane-TB:)

- Nini le?
- Ngaba lo mntu uluthathe unyango kwaye ixesha elingakanani?

Ngaba kukho umntu kusapho lwakho owakhe wane-TB? (Uluhlu lwawo onke amalungu osapho ane-TB:)

- Nini le?
- Ngaba lo mntu uluthathe unyango kwaye ixesha elingakanani?

Ngaba kukho umntu ebumelwaneni bakho okanye emsebenzini onayo okanye one-TB? (uluhlu lwabo bonke abanye abane-TB:)

- Nini le?
- Ngaba lo mntu uluthathe unyango kwaye ixesha elingakanani?

Ngaba ngoku kukho umntu ekhayeni/kusapho/emelwaneni/emsebenzini/ekuhlaleni obekhohlela kangangeeveki ezininzi, ohlile emzimbeni, okanye ogulayo?

UKUHLOLWA KOQHAGAMSHELWANO

Ngaba kukho umntu ekliniki okuchazile malunga nokuhlolwa koqhagamshelwano?

Ingaba ukhona umntu ovela ekliniki oze endlwini yakho eze kuvavanya amalungu ekhaya lakho i-TB?

Ukuba ewe, ngaba uyakhumbula ukuba ngubani oze endlwini yakho? Ingaba ibingumongikazi osuka ekliniki, unompilo wasekuhlaleni, okanye omnye umntu? Ngaba uze yedwa okanye yayiliqela?

Ngaba unokuchaza okwenzekayo ebudeni botyelelo?

Ithathe ixesha elingakanani? Ngaba usayikhumbula imibuzo awayeyibuza?

Ingaba uthathe isikhohlela okanye iisampulu zegazi?

Ukucebisile ngothintelo losulelo ekhaya?

Uye waziva njani malunga nomsebenzi wezempilo oza endlwini yakho? Probe: Ngaba uyifumene iluncedo, iyangenelela, iyafundisa, ibeka amabala, njl

IZIGULO

Ukusuka kwiprofayile yakho yomthathi-nxaxheba, ndiyabona ukuba nawe unayo i-X.

(Ukuba akukho nto ibonisa ezinye iimeko kwiprofayili, ngoko buza: Ngaba unengxaki engapheliyo njenge-HIV, isifo seswekile, uxinzelelo lwegazi oluphezulu?)

Ngaba ungachaza ukuba kunjani ukuba ne-TB kunye nale meko? Phandela amayeza, iziphumo ebezingalindelekanga, ukutyelelwa rhoqo eklinikhi, kunye namanye amalungiselelo

IIGRANTI ZAKWA-SASSA

Ngaba ngoku ufumana isibonelelo? Ukuba kunjalo, sisiphi isibonelelo?

Ngaba usifakile isicelo senkxaso-mali ngelixa ugula yi-TB?

Ngaba unokundichazela inkqubo? Yeyiphi iofisi oya kuyo? Ngawaphi amaxwebhu ekufuneka uhambe nawo? njl

Ngaba ukhona owakuchazelayo ngale nkqubo okanye uyifumene njengoko ubuhamba? Ukuba ewe, ngubani oye wachaza okanye ulufumene phi ulwazi?

Ngaba uyifumene inkqubo elula? Kutheni ewe okanye hayi?

Ithathe ixesha elingakanani yonke le nkqubo?

Ngaba uye waphumelela kwisicelo sakho? Ukuba akunjalo, sasiyintoni isizathu sokukhatywa kwesicelo? Ukuba ewe, ngaba kwamsinyane kangakanani emva kokufaka isicelo usifumene isibonelelo?

Ngokoluvo lwakho, yeyiphi inxalenye yenkqubo yesicelo ebiyeyona inzima/ekukhathazayo kuwe? Kwaye yeyiphi eyona ndawo yayilula?

Ngokoluvo lwakho, ingaphuculwa njani inkqubo yokufaka isicelo?

Ukuba isigulana sifumana isibonelelo, sikuncede njani wena okanye umzi wakho?

<u>ISIPHELO</u>

Phinda kwakhona ngokuyimfihlo "Ndifuna ukukukhumbuza kwakhona ukuba yonke into ebesiyixoxile apha iya kuhlala iphakathi kwam, iqela lophando kolu phononongo kunye nawe. Akukho mntu wangaphandle uya kukwazi ukufikelela kule nkcazelo, yaye igama lakho aliyi kuba kulo naluphi na upapasho neengxelo eziya kubhalwa ngesi sifundo."

Ndiza kukushiyela iphepha lolwazi endikufundele lona ekuqaleni kwale seshini ukuze ukwazi ukulifunda kwakhona ngexesha lakho. Ukuba kukho eminye imibuzo okanye iinkxalabo ezinokuthi zize emva kolu dliwano-ndlebe ndicela uqhagamshelane nam ku-021 938 0821 okanye undithumelele u-"nceda unditsalele" ku-0720478379.

Enkosi....
