# Supplementary material for: “I’m suffering for food”: Food insecurity and access to social protection for TB patients and their households in Cape Town, South Africa
Source: PLoS One. 2022 Apr 26;17(4):e0266356. doi: 10.1371/journal.pone.0266356 (PMC9041827; doi:10.1371/journal.pone.0266356)
Supplement: S2 File — (PDF) [file pone.0266356.s002.pdf]

SUPPORTING INFORMATION FILE 2

TOPIC GUIDE FOR TB PATIENTS - ENGLISH

Before the interview:

- Introduce myself to interviewee, brief explanation of study topic, study purpose "this study seeks to understand the social, economic and household burden of TB by interviewing patients about their experience of TB and the provision of support for TB. The study also seeks to understand patients' experiences of existing health care and social services to support TB patients. "
- Read Information Sheet, reassure confidentiality, signing of consent form
- State expected length of interview: 1 to 2 hour max
- Explain interview process "This interview is really about getting a sense of how you experience being sick with TB, the impact is has on your life, and how you experience accessing health and social services to support you."

History of TB

Please tell me a little about yourself. (Probe for home life, activities, origin, etc)

Have you had TB before?

When was this and did you take treatment at the time? (List all previous episodes + duration of treatment)

Burden of TB

Have you been very sick from TB? Please describe, e.g. were you bedridden and for how long, did you feel tired, lost weight, etc

How else has TB affected your life? (If no answer, probe for the following:

- have you missed time at work? How many days?

- has TB had an impact on the relationship with your household/family/friends?

- has TB had an impact on your life in the community?

Multiple infections in the HH

Who else in your household has had TB? (List for all household members with TB:)

When was this?

Did this person take treatment and for how long?

Has anyone in your family, outside of the household, had TB? (List for all family members with TB:)

When was this?

Did this person take treatment and for how long?

Has anyone in your neighbourhood or at work have or had TB? (list for all others with TB:)

When was this?

Did this person take treatment and for how long?

Is there currently someone in your household/family/neighbourhood/work/community that has been coughing for several weeks, has lost weight, or is sick?

Contact screening

Did anyone at the clinic explain you about contact screening?

Did anyone from the clinic come to your house to test members of your household for TB?

If yes, can you remember who came to your house? Was it a nurse from the clinic, community healthcare worker, or someone else? Did he/she come alone or was it a team?

Can you describe what happened during the visit?

How long did it take? Can you remember the questions he/she asked?

Did he/she take sputum or blood samples?

Did he/she advise you on infection control in the household?

How did you feel about the healthcare worker coming to your house? Probe: Did you find it useful, intrusive, educational, stigmatising, etc

Co- morbidities

From your participant profile, I can see that you also have X.

(If no indication of other conditions on the profile, then ask: Do you suffer from any chronic conditions like HIV, diabetes, high blood-pressure?)

Can you describe what it is like having TB and this other condition? Probe for medication, side-effects, frequency of clinic visits, and other arrangements.

SASSA grants

Are you currently receiving a grant? If yes, which grant?

Have you applied for a grant while being sick with TB?

Can you explain the process for me? Which office do you go to? Which documentation do you need to take with you? Etc

Did anyone explain this process to you or did you find it out as you went along? If yes, who explained or where did you find the information?

Did you find it an easy process? Why yes or no?

How long did the whole process take?

Were you successful in your application? If no, what was the reason for the rejection of the application? If yes, how soon after submitting the application did you receive the grant?

In your opinion, which part of the application process was the most difficult/frustrating for you? And which part was the easiest?

In your opinion, how could the application process be improved?

If the patient is receiving a grant, how has the grant helped you or your household?

End

Reiterate confidentially "I just want to remind you again that everything we have discussed here will remain between me, the research team on this study and yourself. No outside person will have access to this information, and your name will not be on any of the publications and reports that will be written about this study."

I am going to leave the information sheet I read to you at the beginning of this session with you so that you can read it again in your own time. If any other questions or concerns should come up after this interview please feel free to contact me on 021 938 0821 or send me a "please call me" on 0720478379.

Thank you....
