# Supplementary material for: “I’m suffering for food”: Food insecurity and access to social protection for TB patients and their households in Cape Town, South Africa
Source: PLoS One. 2022 Apr 26;17(4):e0266356. doi: 10.1371/journal.pone.0266356 (PMC9041827; doi:10.1371/journal.pone.0266356)
Supplement: S3 File — (PDF) [file pone.0266356.s003.pdf]

SUPPORTING INFORMATION FILE 3

TOPIC GUIDE FOR HEALTHCARE WORKERS ENGLISH

Before the interview:

- Introduce myself to interviewee, brief explanation of study topic, study purpose "this study seeks to understand the burden of TB on patients through interviews with patients and TB nurses."
- Read Information Sheet, reassure confidentiality, signing of consent form
- State expected length of interview: 1 to 2 hour max
- Explain interview process "This interview is really about tapping into your experience as a TB nurse of the burden of TB on a patient and hyow healthcare and social services can assist these patients.

How long have you been working with TB patients?

Question for the SASSA Doctor: What process do you follow in assessing TB patients' eligibility for the CSG?

In your experience, what are the most pressing challenges for TB patients to adhere to their treatment?

Besides treatment, in your opinion, what other support do TB patients need to get better? Probe for provision of food, cash, transport, etc.

What do you think about the provision of social grants for people with chronic conditions including TB?

To your knowledge, what is the process for a patient to apply for a grant? Probe for challenges, gaps,

To your knowledge, how does the referral system between health system and SASSA work? Probe for gaps, challenges,

What role do you play in assisting TB patients applying for a grant?

Regarding the process of applying for a grant, do you think it should be improved? If yes, where do you see room for improvement?

End

Reiterate confidentially "I just want to remind you again that everything we have discussed here will remain between me, the research team on this study and yourself. No outside person will have access to this information, and your name will not be on any of the publications and reports that will be written about this study."

I am going to leave the information sheet I read to you at the beginning of this session with you so that you can read it again in your own time. If any other questions or concerns should come up after this interview please feel free to contact me on 021 938 0821 or send me a "please call me" on 0720478379.

Thank you....
