# Supplementary material for: “I’m suffering for food”: Food insecurity and access to social protection for TB patients and their households in Cape Town, South Africa
Source: PLoS One. 2022 Apr 26;17(4):e0266356. doi: 10.1371/journal.pone.0266356 (PMC9041827; doi:10.1371/journal.pone.0266356)
Supplement: Attachment — Submitted filename: Response to reviewers_251121.pdf [file pone.0266356.s004.pdf]

Dear Editor and Reviewers

Thank you for the opportunity to revise our paper "“I'M SUFFERING FOR FOOD”: FOOD INSECURITY AND ACCESS TO SOCIAL PROTECTION FOR TB PATIENTS AND THEIR HOUSEHOLDS IN CAPE TOWN, SOUTH AFRICA”.

We thank both the editor and the reviewers for their helpful and constructive comments that have allowed us to improve the paper for publication.

Please find our point by point response below. We also include a copy of the revised manuscript with tracked changes to illustrate the changes made, as well as a clean copy.

We hope that these changes have much improved the paper to meet the publishing requirements of PLOSONE.

Sincerely,

Lieve and the research team

COMMENT	RESPONSE
**JOURNAL REQUIREMENTS**	
1. Please ensure that your manuscript meets PLOS ONE's style requirements, including those for file naming. The PLOS ONE style templates can be found at https://journals.plos.org/plosone/s/file?id=wjVg/PLOSOne_formatting_sample_main_body.pdf and https://journals.plos.org/plosone/s/file?id=ba62/PLOSOne_formatting_sample_title_authors_affiliations.pdf	Thank you for providing the style templates. We have revised the manuscript according to the style templates.
2. Please review your reference list to ensure that it is complete and correct. If you have cited papers that have been	Dear editor, we have checked all references on the Retraction Watch Database and none of them returned as retracted.

retracted, please include the rationale for doing so in the manuscript text, or remove these references and replace them with relevant current references. Any changes to the reference list should be mentioned in the rebuttal letter that accompanies your revised manuscript. If you need to cite a retracted article, indicate the article's retracted status in the References list and also include a citation and full reference for the retraction notice.	
3. Please include a copy of the interview guide used in the study, in both the original language and English, as Supporting Information, or include a citation if it has been published previously.	We have included a copy of the interview guide for TB patients in both English and Xhosa, and a copy of the interview guide for healthcare workers in English.
4. Thank you for stating the following financial disclosure: "LV received funding from the South African Medical Research Council for this study. www.samrc.ac.za. The funders had no role in study design, data collection and analysis, decision to publish, or preparation of the manuscript."<br>We note that one or more of the authors is affiliated with the funding organization, indicating the funder may have had some role in the design, data collection, analysis or preparation of your manuscript for publication; in other words, the funder played an indirect role through the participation of the co-authors. If the funding organization did not play a role in the study design, data collection and analysis, decision to publish, or preparation of the manuscript and only provided financial support in the form of authors' salaries and/or research materials, please do the following:<br>a. Review your statements relating to the author contributions, and ensure you have specifically and accurately indicated the role(s) that these authors had in your	**We have reviewed the statement to the following:**<br><br>LV received internal funding from the South African Medical Research Council for this study. The funder provided support in the form of salaries for authors LV and WZ but did not have any additional role in the study design, data collection and analysis, decision to publish, or preparation of the manuscript. The specific roles of these authors are articulated in the 'author contributions' section.

study. These amendments should be made in the online form.<br>b. Confirm in your cover letter that you agree with the following statement, and we will change the online submission form on your behalf:<br>“The funder provided support in the form of salaries for authors [insert relevant initials], but did not have any additional role in the study design, data collection and analysis, decision to publish, or preparation of the manuscript. The specific roles of these authors are articulated in the ‘author contributions’ section.	
5. We note that you have stated that you will provide repository information for your data at acceptance. Should your manuscript be accepted for publication, we will hold it until you provide the relevant accession numbers or DOIs necessary to access your data. If you wish to make changes to your Data Availability statement, please describe these changes in your cover letter and we will update your Data Availability statement to reflect the information you provide.	As we did not seek prior consent from our research participants to make their data publicly available, we are not able to provide access to the data. The SAMRC Human Research Ethics Committee guidelines bar us from making participant data available, even if anonymized, if we did not seek permission and consent from participants during data collection.<br>We therefor request to use the following statement:<br>“The datasets generated and analysed during the current study are not publicly available due to the sensitive nature of the content but are available from the corresponding author on reasonable request.”
6. Your ethics statement should only appear in the Methods section of your manuscript. If your ethics statement is written in any section besides the Methods, please move it to the Methods section and delete it from any other section. Please ensure that your ethics statement is included in your manuscript, as the ethics statement entered into the online submission form will not be published alongside your manuscript.	**The following ethics statement has been added to the Methods section:**<br>**Ethics approval and consent to participate**<br>The study received approval from the Human Research Ethics Committee of the South African Medical Research Council (EC015-82017, October 2017). All participants were given informed consent forms which were read together with the participant and explained in detail before forms were signed. Participants were informed about the purpose of the study, procedures involved, risks and benefits of the study and their rights as participants. The right to decline participation was emphasised, as well as an assurance given that the decision (not) to participate would not affect the healthcare service received at the clinic or repercussions from clinic staff. Participants were given an assurance of confidentiality and strict protection of collected data. The participant characteristics are aggregated to protect participant identities.

<table>
<tr><th colspan="2">REVIEWER 1</th></tr>
<tr><td>1. Wish the authors could add a little material regarding why the patient’s had delayed getting care. Was it fear of income los? Inability to get to the health center easily? This is relevant to social protections for high risk populations and would help target active case finding programs.</td><td>Dear reviewer, thank you for this comment. We looked at the patients’ interviews data again and have made additions to the results and discussion section regarding reasons for the long time between onset of symptoms and diagnosis.

Changes/Additions made to results (line 195-199 and 206-214):

For many participants, however, the time between onset of symptoms and diagnosis of TB was long, i.e. several weeks to several months, resulting in many of them becoming so ill they had to be admitted to hospital.

Reasons for the long time between symptoms and diagnosis were on both the patient and healthcare system side. Patient-related reasons were mostly an underestimation of the cough, thinking it was a flu and treating it with over-the-counter medication. On the side of the health system, about half of the participants mentioned the clinic taking a long time to diagnose. Close to half of the participants mentioned the clinic performing several tests, mostly X-rays, that would return negative. Two participants with a delayed diagnosis had TB of the stomach and TB meningitis respectively which could explain the negative results. Reasons for delayed care-seeking remain an area that requires further investigation

“I would keep coughing and kept coming but they said that they couldn’t see it. I would think but I'm dying, I can feel I'm really coughing, I'm sweating.” (TB-patient, DS-TB, female)

Addition made to discussion (line 367-369):

Patient-related reasons for the delay in diagnosis can be improved with more education and awareness of TB. The facility-related reasons, however, i.e. inability to diagnose timeously, is more concerning and needs further investigation.</td></tr>
<tr><td>2. The idea that the health care workers were not prepared to guide the patients towards disability grants suggests a real need to ensure that TB clinics and</td><td>Dear reviewer, thank you for this comment. We looked at the healthcare worker interviews again and made changes and additions to the results and discussion section regarding their perceptions and experiences of assisting TB patients with the application process for social grants.</td></tr>
</table>

hospitals take on the role of helping patients navigate the arduous task of obtaining grants available to them. This is an important entry point for intervention. How did the healthcare workers interviewed feel about this? Did they feel that it was their responsibility or did they need administrative support for this?	However, we disagree that this is an important entry point for intervention. Patient knowledge about the grant application process and requirements would not help much on its own because access to the grant is doctor-driven. Doctors decide if a person is sick enough and needy enough to ‘’deserve’’ social assistance (line 344-367; line 454-462). We feel that the decision to grant social assistance should not be based on doctors’ discretion. It should be informed by a holistic assessment including other actors involved in TB patient care such as social workers. The Harmonised Tool suggested by DSD a few years ago which never took flight, was a step in this direction. We have added a few lines emphasizing this point in the discussion.<br><br>**Changes/Additions made to results section (line 309-317):**<br><br>Participants generally had little information about the application and assessment process for the DG and reported that they heard about the grant and application process from other TB patients and from family and neighbours. Information about the DG was not generally available in the clinic and participants had to explicitly ask and wait for the doctor or social worker to share the information. Nurses participating in the study said they had no knowledge of the application process and referred patients to the doctor or social worker. Doctors in the study confirmed that they are the main point of information and assistance with the DG application process in the clinic and that this takes up a considerable amount of their time.<br><br>**Changes/addition to discussion (line 431-437):**<br><br>None of the participants in our study, however, received the SRD or food parcel, and few were successful in their application for the DG.<br><br>The application process for the Disability Grant presented many challenges for TB patients. The first challenge is access to information about the application process. Participants in our study testified that information on the DG was not available at the clinic, nor from the nurses. Patients are routinely referred to the doctor as he/she is the one that assesses the patients and makes a recommendation, severely limiting access to information.

	**Addition to discussion (line 452-464):**<br><br>Equally concerning, however, was the finding that despite many regulatory changes to reduce the discretion of the doctor (26), our study showed that doctors performing the medical assessment for the application continue to have high levels of discretion i.e. doctors decide if a person is sick enough and needy enough to ''deserve'' social assistance, allowing doctors' opinions and beliefs such as misuse of grants to influence their assessment. Despite perceptions of misuse of grants such as the Disability Grant, Child Support Grant and the Old Age Grant, there is no evidence supporting this. Social grants, however, have been shown to play an important welfare function in poor households as they are typically shared (53).<br><br>High levels of discretion in assessments for social grants has previously been shown to be a critical issue in assessing people living with HIV to receive the disability grant (54). The Harmonised Assessment Tool (HAT), developed and piloted jointly by the Department of Social Development and the Department of Health, introduced a more holistic and standardized assessment for the DG, reducing the discretion of the doctor, but has never been implemented.
3. Could the authors speak to the validity of the TB doctors concern that disability grant funds would be diverted to luxury items rather than nutrition. Did the use of the disability grant come up in their interviews with the patients?	Thank you. We have added participants' views as to what they would use the financial support on to the results section, as well as a few lines in the discussion section on the evidence regarding misuse of grants.<br><br>**Changes/Additions made to results section (line 352-361):**<br><br>Participants in the study, however, said they would mostly use the money for food, electricity, rent, and the children's needs.<br>"I would be able to give myself energy, buy the foods that are compatible with the treatment. Something that will give me a boost so that I'm alright." (TB patient, DS-TB, male)<br>Several participants, mostly women, also mentioned that it would give them back their independence.<br>"The money helped my sister, because they (household members) received grants but it prioritised their own needs… My mother and my brothers, they received their

	respective grants. .. but now I can go out and buy what I need as well." (TB patient, DS-TB, female)<br>"I'm going to be able to buy it myself and have my own stash (food) at home, where I say okay guys, this cupboard is the sick person's cupboard." (TB patient, DS-TB, female)<br><br>**Addition to discussion (line 452-455):**<br>Despite perceptions of misuse of grants such as the Disability Grant, Child Support Grant and the Old Age Grant, there is no evidence supporting this. Social grants, however, have been shown to play an important welfare function in poor households as they are typically shared.
**REVIEWER 2**	
1. A little more on cash vs. food support will be useful in putting the things into perspective for future researchers and advocacy. Any studies that have explored that in South Africa will be great value. The common arguments are of misuse of cash (as mentioned by the doctor) or food sharing in case of food support (the recurring theme is food in many quotes).	Dear reviewer, thank you for this comment. We have added more information on cash versus food support in South Africa: 1) a paragraph on government provided food parcels (SRD) versus the DG (cash grant) in South Africa to both the results and discussion section, and 2) a few lines on the food versus cash debate in the discussion.<br><br>**Changes/Additions made to results section (line 300-305):**<br><br>In South Africa, social protection in the form of a Disability Grant (DG) or food parcels, also called Social Relief of Distress (SRD), can support patients in the absence of family but also support families to continue caring for patients. None of the participants in our study, however, received the Social Relief of Distress or food parcel and one of the doctors in the study confirmed that<br>"In our experience we find that patients really don't get that-- Sassa always run out of funds to be able to help patients who require that social relief assistance." (TB doctor)<br><br>**Addition to discussion (line 427-431):**<br><br>Effectiveness and efficiency of food versus cash has, to our knowledge, not been investigated in the South African context, yet research conducted in sub-Saharan African, Asian and South-American countries has shown that both cash transfers and food aid are effective but cash transfers and vouchers tend to be more efficient and less costly than food-based interventions.

2. A point in discussion to emphasize the support to family rather than just the patient. Especially considering the renewed focus on households in National Strategic Plan of South Africa for HIV, TB and STIs 2017-2022.	Thank you. We have added a paragraph on the focus on households in the NSP to the discussion.<br><br>**Addition to discussion (line 423-427):**<br>The National Strategic Plan for HIV, TB and STI 2017-2022 recognises the vital role of households in supporting TB patients and the need to empower and strengthen households as key actors. The Department of Social Development is to play the lead role in building strong social support systems and has several tools to its disposal to support TB patients and their households such as the Social Relief of Distress (SRD) or food parcel and the Disability Grant (DG).
3. This was done in 2017-18 and we are now in 2021. It will be a good idea to put this limitation upfront and mention the newer developments or the lack of it, in SDoH/social protection with reference to TB in the country.	Thank you. We have added a paragraph to the strengths and limitations section explaining that the data was collected in 2017-2018, nearly 4 years ago. However, we must also make note that this manuscript was submitted to PLOSONE in March 2021.<br><br>**Addition to discussion (line 472-478):**<br>A second limitation is that the interviews were conducted in 2017-2018, nearly 4 years ago. Nevertheless, the challenges presented in this article remain relevant and have to date not been resolved nor addressed. In addition, there has been no change in the social grants policy for TB patients in South Africa.
4. Discuss the findings of this study and lack of evidence for support for patients (nutrition and others) as found by 10.1002/14651858.CD007952.pub3; 10.1002/14651858.CD006086.pub4	Thank you. We included a few lines on the evidence regarding food and/or case support, including the suggested articles (Lutge et al 2015, Grobler et al 2016) as well as more recent evidence (Wingfield et al 2017, Watthananukul et al 2020).<br><br>**Addition to discussion (line 419-423):**<br>While there is limited evidence on the effect of food supplementation and financial support on treatment outcomes for TB patients (49, 50), financial support has been shown to positively impact on TB risk factors (16) increase treatment success (17), improve clinic attendance (50) and reduce out-of-pocket payments for TB patients (19).
